# Inhibition of the ATP Synthase Eliminates the Intrinsic Resistance of *Staphylococcus aureus* towards Polymyxins

**DOI:** 10.1128/mBio.01114-17

**Published:** 2017-09-05

**Authors:** Martin Vestergaard, Katrine Nøhr-Meldgaard, Martin Saxtorph Bojer, Christina Krogsgård Nielsen, Rikke Louise Meyer, Christoph Slavetinsky, Andreas Peschel, Hanne Ingmer

**Affiliations:** a Department of Veterinary Disease Biology, Faculty of Health and Medical Sciences, University of Copenhagen, Frederiksberg C, Denmark; b Interdisciplinary Nanoscience Center, Aarhus University, Aarhus C, Denmark; c Department of Bioscience, Aarhus University, Aarhus C, Denmark; d Interfaculty Institute of Microbiology and Infection Medicine, Infection Biology Section, University of Tübingen, and German Center for Infection Research (DZIF), Partner Site Tübingen, Tübingen, Germany; Ramón y Cajal University Hospital; Harvard Medical School

**Keywords:** ATP synthase, *Staphylococcus aureus*, antimicrobial peptides, *atpA*, intrinsic resistance, oligomycin A, polymyxin

## Abstract

*Staphylococcus aureus* is intrinsically resistant to polymyxins (polymyxin B and colistin), an important class of cationic antimicrobial peptides used in treatment of Gram-negative bacterial infections. To understand the mechanisms underlying intrinsic polymyxin resistance in *S. aureus*, we screened the Nebraska Transposon Mutant Library established in *S. aureus* strain JE2 for increased susceptibility to polymyxin B. Nineteen mutants displayed at least 2-fold reductions in MIC, while the greatest reductions (8-fold) were observed for mutants with inactivation of either *graS*, *graR*, *vraF*, or *vraG* or the subunits of the ATP synthase (*atpA*, *atpB*, *atpG*, or *atpH*), which during respiration is the main source of energy. Inactivation of *atpA* also conferred hypersusceptibility to colistin and the aminoglycoside gentamicin, whereas susceptibilities to nisin, gallidermin, bacitracin, vancomycin, ciprofloxacin, linezolid, daptomycin, and oxacillin were unchanged. ATP synthase activity is known to be inhibited by oligomycin A, and the presence of this compound increased polymyxin B-mediated killing of *S. aureus*. Our results demonstrate that the ATP synthase contributes to intrinsic resistance of *S. aureus* towards polymyxins and that inhibition of the ATP synthase sensitizes *S. aureus* to this group of compounds. These findings show that by modulation of bacterial metabolism, new classes of antibiotics may show efficacy against pathogens towards which they were previously considered inapplicable. In light of the need for new treatment options for infections with serious pathogens like *S. aureus*, this approach may pave the way for novel applications of existing antibiotics.

## IMPORTANCE

Bacterial pathogens that cause disease in humans remain a serious threat to public health, and antibiotics are still our primary weapon in treating bacterial diseases. The ability to eradicate bacterial infections is critically challenged by development of resistance to all clinically available antibiotics. Polymyxins constitute an important class of antibiotics for treatment of infections caused by Gram-negative pathogens, whereas Gram-positive bacteria remain largely insusceptible towards this class of antibiotics. Here we performed a whole-genome screen among nonessential genes for polymyxin intrinsic resistance determinants in *Staphylococcus aureus*. We found that the ATP synthase is important for polymyxin susceptibility and that inhibition of the ATP synthase sensitizes *S. aureus* towards polymyxins. Our study provides novel insights into the mechanisms that limit polymyxin activity against *S. aureus* and provides valuable targets for inhibitors to potentially enable the use of polymyxins against *S. aureus* and other Gram-positive pathogens.

## INTRODUCTION

Polymyxins (polymyxin B [PMB] and colistin) are lipopeptide antibiotics that consist of a peptide ring with a three-peptide side chain linked to a fatty acid tail. At physiological pH, polymyxins are polycationic, which in combination with the fatty acid tail makes them amphipathic. The amphipathic property of polymyxins promotes interaction with cell membranes, eventually leading to disruption of membrane integrity and cell death (1). The spectrum of activity of polymyxins is primarily confined to Gram-negative bacteria (2), where they increase the permeability of the outer membrane and the cytoplasmic membrane (3). Reduced susceptibility to polymyxins in Gram-negative bacteria can be mediated by reduction of the negative cell surface charge, which limits the electrostatic interaction between the positively charged polymyxins and negatively charged lipopolysaccharides (4).

Polymyxins are generally less active against Gram-positive bacteria (2), and *Staphylococcus aureus* is intrinsically resistant to PMB and colistin (5). The mechanisms conferring intrinsic resistance to polymyxins are not completely understood. However, the sensitivity of *S. aureus* to structurally different cationic antimicrobial peptides has been demonstrated to be affected through proteolytic degradation of the human cathelicidin LL-37 by the protease aureolysin, sequestration of human α-defensins by staphylokinase, alterations of cell surface charge, and active efflux of tPMP-1 (thrombin-induced platelet microbicidal protein 1) by the efflux pump QacA (4, 6–8).

In *S. aureus*, two mechanisms have been demonstrated to alter cell surface charge in response to the presence of cationic antimicrobial peptides (9, 10). Incorporation of d-alanine on teichoic acids, mediated by the *dltABCD* operon, reduces the net negative charge of the cell surface and thereby reduces electrostatic interaction with cationic antimicrobial peptides (9). Similarly, incorporation of l-lysine to membrane phosphatidylglycerols by the enzyme MprF (FmtC) also reduces the net negative charge (10). Regulation of the *dlt* operon and *mprF* expression is mediated *via* the three-component system GraXSR (also known as ApsXSR), which together with the VraFG transporter system can sense and signal the presence of cationic antimicrobial peptides (6, 11). Inactivation of *graR* and *vraG* has previously been shown to increase the susceptibility of *S. aureus* to PMB (12), whereas degradation and sequestration have not been reported to affect polymyxin susceptibility in *S. aureus*.

*S. aureus* is an opportunistic human pathogen that can cause a variety of diseases ranging from skin infections to life-threatening systemic infections (13). The slow introduction of novel antimicrobial molecules to the clinic necessitates the understanding of the determinants that make *S. aureus* intrinsically resistant to polymyxins (14), an antimicrobial class that is extensively used against Gram-negative infections (1). Knowledge of intrinsic resistance mechanisms could provide targets for helper drugs to sensitize *S. aureus* to polymyxins. Therefore, we screened the Nebraska Transposon Mutant Library (NTML) of 1,920 single-gene inactivations in *S. aureus* JE2 for mutants (15), which were unable to grow at subinhibitory concentrations of PMB. The screen revealed multiple novel polymyxin intrinsic resistance genes, most importantly genes encoding subunits of the ATP synthase.

## RESULTS

### The polymyxin B intrinsic resistome.

*Staphylococcus aureus* is intrinsically resistant to the clinically approved cationic antimicrobial peptides polymyxin B (PMB) and colistin (1, 16). To identify intrinsic resistance mechanisms in *S. aureus*, we screened the entire NTML for mutants that displayed lack of growth on agar plates supplemented with PMB equal to 0.5× the MIC of the wild type (WT). The MIC for PMB was subsequently determined using Etests for the identified mutants. Nineteen mutants were confirmed to be at least 2-fold more susceptible than the WT (Table 1). As expected, we identified transposon insertions in *graS*, *graR*, *vraF*, and *vraG*, corroborating previous work on these determinants in *S. aureus* in relation to increased PMB susceptibility (12). Furthermore, transposon insertion in the potassium transporter gene* trkA* had a minor effect on PMB susceptibility, as previously observed in *Vibrio vulnificus* (17).

**TABLE 1:** Intrinsic polymyxin B resistance determinants identified in the NTML and the corresponding MICs of polymyxin B and colistin

Gene name	Function	Gene no.	MIC (µg/ml)	
			Polymyxin B	Colistin
Wild type (*S. aureus* JE2)			128	>256
*vraG*	ABC transporter, permease protein	*SAUSA300_0648*	16	24
*vraF*	ABC transporter, ATP-binding protein	*SAUSA300_0647*	16	32
*graR*	DNA-binding response regulator	*SAUSA300_0645*	16	32
*graS*	Sensor histidine kinase	*SAUSA300_0646*	24	32
*atpA*	ATP synthase F_1_, α subunit	*SAUSA300_2060*	16	48
*atpB*	F_o_F_1_ ATP synthase subunit A	*SAUSA300_2064*	16	48
*atpG*	F_o_F_1_ ATP synthase subunit γ	*SAUSA300_2059*	16	48
*atpH*	F_o_F_1_ ATP synthase subunit δ	*SAUSA300_2061*	16	48
*cbiO*	Cobalt transporter ATP-binding subunit	*SAUSA300_2176*	48	64
*trkA*	Potassium uptake protein	*SAUSA300_0988*	48	128
	Putative ABC transporter protein EcsB	*SAUSA300_1785*	48	>256
*vraS*	Two-component sensor histidine kinase	*SAUSA300_1866*	64	256
*yajC*	Preprotein translocase subunit YajC	*SAUSA300_1594*	64	256
*lspA*	Lipoprotein signal peptidase	*SAUSA300_1089*	64	256
	Diacylglycerol glucosyltransferase	*SAUSA300_0918*	64	>256
	Hypothetical protein	*SAUSA300_1802*	64	>256
	Hypothetical protein	*SAUSA300_1254*	48	192
	Hypothetical protein	*SAUSA300_0980*	48	>256
	Hypothetical protein	*SAUSA300_1495*	64	>256

Interestingly, inactivation of multiple genes encoding subunits of the ATP synthase displayed increased sensitivity towards polymyxins: the genes included *atpA*, *atpB*, *atpG*, and *atpH*. The ATP synthase generates ATP from ADP and P_i_ at the F_1_ domain with energy derived from proton movement through the F_o_ domain (18). The F_1_ domain is an assembly of five proteins with the stoichiometry α_3_β_3_γ_1_δ_1_ε_1_ (18), where *atpA* encodes the α-subunit, *atpG* encodes the γ-subunit, and *atpH* encodes the δ-subunit. The gene *atpB* encodes the A-subunit of the F_o_ domain. ATP catalysis proceeds at the β-subunits, whereas the functions of the α-subunits remain poorly understood, but have been shown to be important for attaining maximum activity of the ATP synthase (19). To the best of our knowledge, the ATP synthase has not previously been associated with PMB sensitivity in Gram-positive bacteria. However, in Gram-negative bacteria such as *Escherichia coli*, inactivation of *atpG* increased sensitivity towards colistin (20), in *Proteus mirabilis*, a mutant with inactivation of a gene with similarity to one of the ATP synthase genes displayed increased sensitivity to PMB (21), and in *Vibrio parahaemolyticus*, antimicrobial peptide-resistant mutants displayed upregulation of the ATP synthase F_1_ α-subunit (22).

While we were unable to complement the *atpA*-inactivated mutant with a functional *atpA* gene on a plasmid, we successfully performed allelic exchange of the transposon insertion with the intact *atpA* gene, generating a strain displaying PMB sensitivity like that of the WT (data not shown).

The remaining mutants identified in the screen only displayed minor increases in PMB susceptibility (Table 1). For all of the mutants displaying increased susceptibility to PMB in the NTML, we additionally measured the susceptibility to colistin. Colistin was less effective against *S. aureus* JE2 than PMB; however, increased sensitivity to PMB correlated with increased sensitivity to colistin (Table 1).

### Medium composition affects the absolute MIC.

It has been reported that growth medium composition can affect polymyxin MIC (23): therefore, we also tested polymyxin B MICs of the WT and *atpA*, *graR*, *vraG*, and *vraF* mutants by employing the Etest on cation-adjusted Mueller-Hinton (MH) agar. Polymyxin B displayed greater activity against *S. aureus* on MH agar than on tryptic soy agar (TSA) plates; however, the fold changes between the WT and mutants largely remain identical (see Table S1 in the supplemental material). Interestingly, strains with inactivation of *atpA*, *graR*, *vraG*, and *vraF* are around the breakpoint level of being susceptible according to the guidelines of CLSI (1, 24). Polymyxin B breakpoints for *Pseudomonas aeruginosa* are susceptible at a MIC of ≤2 μg/ml, intermediate at a MIC of 4 μg/ml, and resistant at a MIC of ≥8 μg/ml, whereas for *Acinetobacter* spp., a MIC of ≥4 μg/ml is considered resistant.

10.1128/mBio.01114-17.1 TABLE S1  Polymyxin B susceptibility of the WT and selected mutants when assayed on cation-adjusted Mueller-Hinton agar plates. Download TABLE S1, DOCX file, 0.1 MB. Copyright © 2017 Vestergaard et al. 2017 Vestergaard et al. This content is distributed under the terms of the Creative Commons Attribution 4.0 International license.

### d-Alanylation of teichoic acids and lysinylation of phosphatidylglycerols.

No mutants with inactivation of genes in the* dltABCD* operon exist in the NTML (15); however, incorporation of d-alanine on teichoic acids mediated by the *dltABCD* operon has previously been revealed to affect susceptibility to cationic antimicrobial peptides (9). Therefore, we examined PMB susceptibility of an isogenic strain pair, namely, an SA113 Δ*dltA* mutant (PMB MIC, 48 µg/ml) relative to the WT parent strain, SA113 (PMB MIC, 512 µg/ml) (see Table S2 in the supplemental material). The result shows that d-alanylation of teichoic acids also is mediating resistance to PMB.

10.1128/mBio.01114-17.2 TABLE S2  Polymyxin B susceptibility of the WT (SA113) and SA113 Δ*dltA* mutant (as no *dlt* mutants exist in the NTML) and SA113 Δ*mprF* mutant (to confirm the unchanged susceptibility towards polymyxins, as seen for the NTML transposon *mprF* mutant). Download TABLE S2, DOCX file, 0.1 MB. Copyright © 2017 Vestergaard et al. 2017 Vestergaard et al. This content is distributed under the terms of the Creative Commons Attribution 4.0 International license.

By screening the NTML, the mutant with inactivation of *mprF* did not display increased sensitivity to PMB, and to confirm this result, we tested PMB susceptibility of the SA113 Δ*mprF* mutant (PMB MIC, 384 µg/ml) relative to ancestral WT strain SA113 (PMB MIC, 512 µg/ml) (Table S2). This suggests that lysinylation of phosphatidylglycerol is not an intrinsic PMB resistance mechanism, in contrast to other classes of cationic antimicrobial peptides (10, 25). Lysinylation of phosphatidylglycerols therefore seems to mediate selective protection against certain cationic antimicrobial peptides.

### Inactivation of *atpA* confers hypersusceptibility to gentamicin.

We assessed whether an impaired ATP synthase affected susceptibility to other antimicrobial peptides (Table 2) and conventional antimicrobial agents (Table 3) by comparing the *atpA* mutant with the WT, as well as the established determinants *vraG*, *vraF*, and *graR*. Only marginal changes in susceptibility to the antimicrobial peptides bacitracin, gallidermin, and nisin, were detected for all of the mutants (Table 2). The *vraF* and *graR* mutants displayed at least 2-fold increased sensitivity to the human cathelicidin LL-37, whereas *atpA* was indistinguishable from the WT (Table 2). For conventional antimicrobial agents, minor reductions in vancomycin MIC were observed for *vraG*, *vraF*, and *graR* mutants, but not for the *atpA* mutant. The *vraG*, *vraF*, and *graR* mutants displayed increased sensitivity to gentamicin (3- to 6-fold), whereas *atpA* displayed a 16-fold increased sensitivity (Table 3). Contrarily, no differences in sensitivities between all the mutants and the WT were detected for ciprofloxacin, linezolid, oxacillin, and daptomycin (Table 3). Increased susceptibility of the *atpA* mutant was restricted to polymyxins and aminoglycosides, demonstrating that the ATP synthase is not generally involved in reducing antimicrobial activity of cationic antibiotics or antimicrobial peptides.

**TABLE 2:** MICs of antimicrobial peptides for the wild type and selected mutants

Peptide	Charge	MIC (µg/ml)				
		WT	*vraG*	*atpA*	*vraF*	*graR*
Gallidermin	Cationic	16	8	16	8	8
Nisin	Cationic	512	256	256	256	256
LL-37	Cationic	>128	128	>128	64	64
Bacitracin	Neutral	256	256	128	256	128

**TABLE 3:** MICs of conventional antibiotics for the wild type and selected mutants

Antibiotic	Charge	MIC (µg/ml)				
		WT	*vraG*	*atpA*	*vraF*	*graR*
Ciprofloxacin	Neutral	32	32	32	32	32
Oxacillin	Anionic	0.5	0.50	0.500	0.50	0.50
Linezolid	Neutral	2	2	2	2	2
Gentamicin	Cationic	1.5	0.25	0.094	0.38	0.50
Vancomycin	Cationic	1.5	1	1.500	0.75	1
Daptomycin	Cationic	0.25	0.25	0.250	0.25	0.19

### The *atpA* mutant displays hyperpolarization of the membrane.

The magnitude of the membrane potential can have a large effect on the activity of antimicrobial peptides against different bacterial species (26). It has been hypothesized that due to the negative orientation of the membrane potential, cationic antimicrobial peptides are electrophoretically drawn into the nonpolar membrane (26). Furthermore, uptake of gentamicin into the cell is dependent on membrane potential, where hyperpolarization of the membrane increases uptake, while depolarization reduces uptake (27).

**FIG 1:**
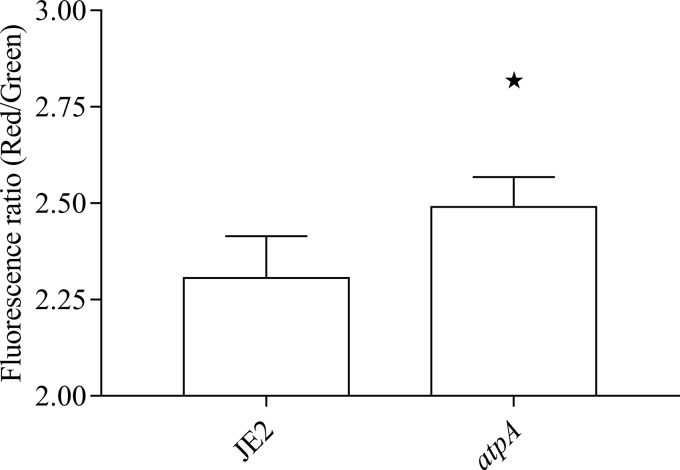
Membrane potentials of the WT (JE2) and *atpA* mutant when assayed with the fluorescent dye DiOC_2_ (3). The *atpA* mutant displayed hyperpolarization of the membrane after 5 min of staining. The data represent the average from three measurements, with errors bars showing 95% confidence intervals. The black star indicates significant difference at *P* < 0.05.

We therefore hypothesized that the *atpA* mutant was more susceptible to PMB due to hyperpolarization of the membrane in the absence of ATP synthase activity. Hence, we assessed the membrane potential for the *atpA* mutant using the fluorescent dye DiOC_2_ (3), and indeed, the *atpA* mutant displayed hyperpolarization of the membrane (Fig. 1). This corroborates a previous study on an ATP synthase-deficient ΔF_o_F_1_ mutant strain of *Corynebacterium glutamicum*, which also displayed increased membrane potential relative to the wild type (28).

### Cell surface charge remains unchanged for the *atpA* mutant.

**FIG 2:**
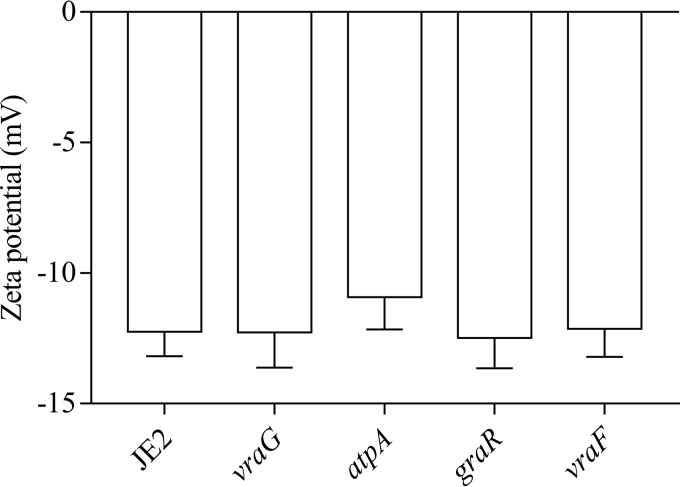
Zeta potential of the WT (JE2) and selected mutants. No significant changes in zeta potential were detected between the wild type and tested mutants. The data represent the average from six measurements, with errors bars showing 95% confidence intervals.

**FIG 3:**
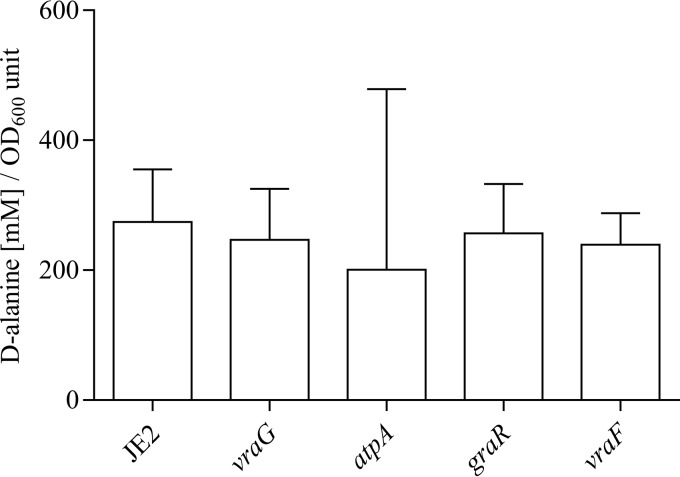
d-Alanylation of teichoic acids. No statistical difference on d-Ala content in teichoic acids between the WT (JE2) and selected mutants. The data represent the average from three measurements, with error bars showing 95% confidence intervals.

A change toward a less negative cell surface charge has previously been correlated with a decrease in susceptibility to cationic antimicrobial peptides (9, 29–31). To assess the potential correlation between cell surface charge and sensitivity to PMB in our mutants, we measured the zeta potential of the *atpA*, *vraG*, *graR*, and *vraF* mutants and the WT (Fig. 2). No significant changes in zeta potentials were detected. Furthermore, we could not detect any significant differences between the *atpA* mutant and the WT for d-alanine content on teichoic acids (Fig. 3) or for the relative content of lysinylated phosphatidylglycerols in the membrane (data not shown).

### Inhibition of the ATP synthase increases efficacy of polymyxin B.

**FIG 4:**
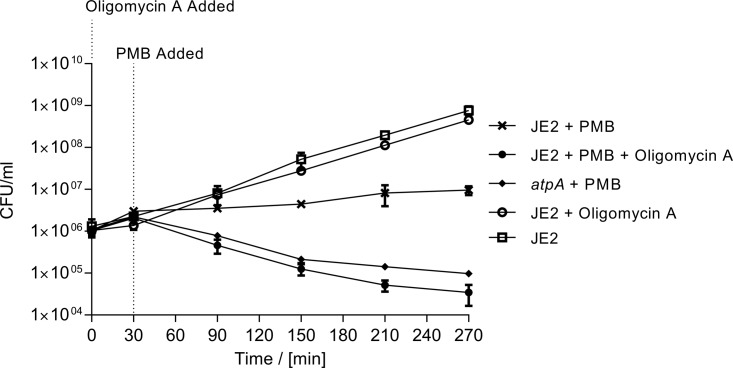
Improved killing efficacy of PMB upon inhibition of the ATP synthase. Antibacterial activities of polymyxin B (0.25× MIC) alone or in combination with the ATP synthase inhibitor oligomycin A (8 μg/ml) were assayed against the WT. As a control of the target, the killing efficacy of polymyxin B (0.25× MIC) was determined for the *atpA* mutant. The data represent the average from three measurements, with error bars showing 95% confidence intervals.

The ATP synthase is a well-described protein complex, and multiple inhibitors have been identified that interfere with its function—e.g., the macrolide oligomycin A (32). To demonstrate the potential of the ATP synthase as a target for potentiating the efficacy of polymyxins against *S. aureus*, we assessed the killing efficacy of PMB in the presence or absence of the ATP synthase inhibitor oligomycin A (Fig. 4). At a concentration of PMB equal to 0.25× the MIC of the WT, the combination therapy (PMB plus oligomycin A) reduced the colony-forming units (CFU) 60-fold after 4 h for the WT, whereas continued growth was observed for WT with treatment with PMB alone. The combinatory efficacy of PMB and oligomycin A is similar to the observed killing efficacy of PMB against the *atpA* mutant. Oligomycin A alone at the provided concentration (8 μg/ml) did not display any killing efficacy against *S. aureus*.

## DISCUSSION

The limited availability of effective and well-tolerated therapies for antibiotic-resistant *S. aureus* has led to a search for inhibitors to improve the efficacy of existing antibiotic compounds by targeting acquired and intrinsic resistance mechanisms (33–36). Inhibition of wall teichoic acid synthesis restored β-lactam efficacy against methicillin-resistant *S. aureus* (33) and fluoroquinolone efficacy was increased by inhibition of the efflux pump NorA (35, 36). These studies, however, have focused on potentiating the efficacy of antibiotics that are normally used against staphylococcal infections and have not included antibiotics that *S. aureus* is intrinsically resistant against.

The present study provides the first whole-genome overview of intrinsic polymyxin resistance genes in *S. aureus*. Most importantly, we identified the ATP synthase as a novel target for potentiating the efficacy of polymyxins against *S. aureus*. Inhibition of the ATP synthase potentiates equally well the efficacy of polymyxins against *S. aureus* as inactivation of the previously established two-component system GraSR and the VraFG transporter system (Table 1).

The bacterial ATP synthase has been validated as an antimicrobial target with the recent approval of the antituberculosis agent bedaquiline (14). Bedaquiline selectively targets the subunit c of the ATP synthase in most mycobacteria, while displaying limited or no activity against other bacterial pathogens, including *S. aureus* (37). Derivatives of the diarylquinoline scaffold of bedaquiline have been generated to increase the activity towards other important Gram-positive pathogens (e.g., *S. aureus*), while still displaying limited or no activity against Gram-negative bacteria (38). Chemical inhibition of the ATP synthase with oligomycin A significantly increased the antistaphylococcal activity of PMB (Fig. 4). However, oligomycin A is nonselective and therefore also inhibits the mitochondrial ATP synthase (38), rendering it inappropriate for human use. Numerous other compounds have been identified that interact with ATP synthases (32), which can be explored as potentiators of polymyxins and aminoglycosides in *S. aureus* for human use. The ATP synthase also constitutes a potential target for potentiation of polymyxins against Gram-negative bacteria, as inactivation of *atpG* in *E. coli* increased sensitivity towards colistin (20).

Inactivation of the ATP synthase conferred hyperpolarization of the membrane (Fig. 1), and we propose this as a potential mechanism for the improved activity of polymyxins. Increased membrane potential may correlate with increased activity of other cationic antimicrobial peptides (26). Furthermore, deletion of the gene *phoP* in *E. coli* conferred hyperpolarization of the membrane and a concomitant increase in activity of PMB, while collapsing the proton gradient with *m*-chlorophenyl carbonyl cyanide hydrozone (CCCP) abrogated this effect (39).

The spectrum of activity of polymyxins also indicates the interrelatedness of the electron transport chain with polymyxin activity, as polymyxins generally display bactericidal activity against Gram-negative bacteria, except against anaerobic Gram-negative bacteria (23, 40, 41). The killing efficiency of polymyxins against *P. aeruginosa* has been reported to be diminished under anaerobic compared to aerobic conditions (42); however, another study could not confirm this (43).

The interrelatedness of the ATP synthase and membrane potential with polymyxin susceptibility is not yet completely understood; however, we have demonstrated that the ATP synthase is a potential target for sensitizing *S. aureus* towards polymyxins. The ATP synthase may also be targeted for potentiating the efficacy of aminoglycosides and potentially other cationic antimicrobial peptides not tested in this study.

Taken together, a greater understanding of the mechanisms conferring intrinsic resistance can provide novel targets for development of inhibitors to potentiate the efficacy of polymyxins and thereby potentially broaden the spectrum of activity of this class of antibiotics to important Gram-positive pathogens. With the need for new treatment options for infections with serious pathogens like *S. aureus*, targeting intrinsic resistance mechanisms may pave the way for novel applications of existing antibiotics.

## MATERIALS AND METHODS

### Bacterial strains, growth conditions, and MIC determination.

The strains used in this study include *S. aureus* strain JE2 (plasmid-cured derivative of USA300 LAC) and all derivative strains within the Nebraska Transposon Mutant Library (NTML), consisting of 1,920 unique transposon mutants with inactivation of nonessential genes (15). The *bursa aurealis* transposon used to create the collection contains the resistance cassette *ermB*, which confers resistance to erythromycin (15). Additionally we used *S. aureus* SA113 and two derivatives, SA113 Δ*dltA* (9) and SA113 Δ*mprF* (10). All bacterial strains were cultured at 37°C in tryptic soy broth (TSB) or on tryptic soy agar (TSA), with antimicrobial agents added as indicated. Two methods have been employed to determine MICs to various antimicrobial agents. (i) A 2-fold broth microdilution assay in TSB (100 µl) with an initial inoculum of approximately 5 × 10^5^ cells/ml was employed to determine the MICs of polymyxin B sulfate (Sigma), gallidermin (Santa Cruz Biotechnology), nisin (Sigma), bacitracin (Sigma), and LL-37 (Isca Biochemicals). (ii) An Etest (BioMérieux) performed on TSA plates was employed to determine MICs for polymyxin B, colistin, ciprofloxacin, oxacillin, linezolid, gentamicin, vancomycin, and daptomycin. The MIC was determined upon incubation at 37°C for 22 h. When indicated, the Etest (BioMérieux) was performed on Mueller-Hinton agar plates (cation adjusted for calcium and magnesium).

### Screening for increased polymyxin B susceptibility.

The NTML is stored in glycerol at −80°C in 20 96-well microtiter plates. Material from the frozen stock was transferred directly with a Deutz 96 cryoreplicator (44) from the 96-well microtiter plates onto TSA plates supplemented with 5 µg/ml erythromycin (as all the strains in the NTML are resistant to erythromycin [15]) and 64 µg/ml polymyxin B (0.5× the MIC). The plates were incubated at 37°C for 24 h and visually inspected for lack of growth of individual mutants.

### Zeta potential.

Overnight cultures were incubated at 37°C with orbital shaking at 180 rpm, harvested by centrifugation at 4,600 × *g* for 10 min, and suspended in phosphate-buffered saline (PBS: 10 mM phosphate buffer, 2.7 mM potassium chloride, 137 mM sodium chloride) to a density of 2.8 × 10^8^ to 4.6 × 10^8^ cells/ml. Zeta potentials were measured at 25°C with a Zetasizer Nano ZS (Malvern Instruments) using folded capillary cells (Malvern Instruments). Six measurements were taken for each sample, and zeta potentials were calculated using the Smoluchowski equation with Zetasizer software (v7.02).

### Assessment of membrane potential measurements using flow cytometry.

Membrane potential was assessed using a flow cytometry assay based on the BacLight bacterial membrane potential kit (Life Technologies). Cells from overnight cultures were inoculated in 10 ml TSB in 100-ml Erlenmeyer flasks and grown to an optical density at 600 nm (OD_600_) of 0.2. Fifteen microliters of culture was transferred to 1 ml filtered PBS. To each cell solution, 10 μl of the fluorescent membrane potential indicator dye DiOC_2_ (3) was added and cells were stained for 5 min at room temperature. Data were recorded on a BD Biosciences Accuri C_6_ flow cytometer (Becton, Dickinson and Company), with emission filters suitable for detecting red and green fluorescence. Settings on the flow cytometer were as follows: 50,000 recorded events at a forward scatter (FSC) threshold of 15,000 and medium flow rate. Gating of the stained cell population and analysis of flow cytometry data were performed in CFlow (BD Accuri). As an indicator of membrane potential, the ratio of red to green fluorescence intensity was calculated. The assay was verified with the NTML mutant containing a transposon insertion in *menD* (NE1345), which displays depolarization of the membrane (45).

### Chromosomal reconstruction of *atpA.*

Chromosomal reconstruction of the *atpA* mutant was achieved by use of the temperature-sensitive shuttle vector pBASE6 (46). A chromosomal region encompassing *atpA* was PCR amplified from WT *S. aureus* JE2 chromosomal DNA using primer pair 5′-ATATGAGCTCGAAGAGTTAGATAAGATTGTCAAACTAG-3′ and 5′-GATACAAGATCTGATGGTTTGTATTGCTACTTGC-3′ and cloned into pBASE6 via SacI/BglII. This plasmid was purified from *E. coli* IM08B (47) and transformed directly into JE2 *atpA*::ΦNΣ (NE592) at 30°C followed by chromosomal integration by plating on TSA (10 µg/ml chloramphenicol) at 44°C overnight. Plasmid cross-out was performed by passage at 30°C followed by plating on TSA (500 ng/ml anhydrotetracycline), and successful allelic exchange of the transposon insertion with the intact *atpA* gene was selected for by replica plating of colonies and screening for sensitivity toward erythromycin and chloramphenicol. Reconstruction of the *atpA* locus was verified by PCR amplification using primers 5′-CAAGTATGCTAAAGCATTATTTGACGTGTC-3′ and 5′-CGTAATTTCTGCTTGTCTCGCTCTG-3′ positioned outside the chromosomal region used for homologous recombination.

### Kill curve experiment assessing polymyxin B efficacy upon inhibition of the ATP synthase.

From overnight cultures of *S. aureus* JE2 and the derivative *atpA* mutant, 100 μl was diluted into 900 μl fresh TSB medium in a Falcon tube and grown for 1 h for the cells to reach the early exponential phase. After 1 h, the cultures were diluted into 10 ml fresh TSB medium in 100-ml Erlenmeyer flasks, reaching an initial cell count of approximately 10^6^ cells/ml. Oligomycin A (Sigma) was added to flasks as indicated at a concentration of 8 μg/ml. After 30 min of growth, polymyxin B was added to flasks as indicated at a concentration equal to 0.25× the MIC. CFU were determined on TSA plates before addition of oligomycin A (time zero [*T*0]), before addition of polymyxin B (time 30 min [*T*_30_]), and every hour for the following 4 h.

### Analysis of d-alaninylation of the *S. aureus* cell envelope.

*S. aureus* JE2 and the mutant strains were grown to the early stationary phase (6 h), washed with ammonium acetate buffer (20 mM), and adjusted to an OD_600_ of 30 in a total volume of 1 ml. Cells were taken up in NaOH to a final volume of 100 μl and were incubated for 1 h of shaking at 37°C to hydrolyze the d-alanine esters. The reaction was stopped with 100 μl of HCl, and the precipitated cell debris was removed by centrifugation and sterile filtration. d-Alanine was derivatized with *ortho*-phthaldialdehyde (OPA), similar to previous experiments (48). Five microliters OPA and 5 µl substrate were mixed for 120 s, and the reaction was stopped by adding 3 µl 100% acetic acid. The sample was then separated via ultraperformance liquid chromatography (UPLC) with an Acquity H class UPLC system from Waters. Five microliters sample was run on a gradient in 24 min from 100% buffer A (25 mM sodium phosphate buffer, pH 7.2) to 100% buffer B (45% acetonitrile, 45% methanol, 10% H_2_O) in a stepwise manner. The column temperature was 23°C, and the flow rate was 0.32 ml/min. Fluorescence was detected at 338 nm.

### Isolation and quantification of polar lipids.

Polar lipids were isolated from *S. aureus* cultures grown to the logarithmic phase (OD_600_ of 0.8) and extracted with chloroform-methanol-sodium acetate buffer (20 mM) (1:1:1 by volume) by the Bligh-Dyer method (49), vacuum dried, and dissolved in chloroform-methanol (2:1 by volume). Amino group- or phosphate group-containing lipids were detected by ninhydrin or molybdenum blue staining, respectively. Aminoacyl phospholipids were quantified in relation to total phospholipid content by determining lipid spot intensities of molybdenum blue-stained lipids as described recently (50).

### Statistics.

The data were analyzed in GraphPad Prism 7 (GraphPad Software, Inc.) using one-way analysis of variance (ANOVA) with a *post hoc* analysis of Dunnett’s multiple comparison tests, where *P* < 0.05 is considered significant (highlighted with a black star in Fig. 1).
