# Supplementary material for: Inhibition of the ATP Synthase Eliminates the Intrinsic Resistance of Staphylococcus aureus towards Polymyxins
Source: mBio. 2017 Sep 5;8(5):e01114-17. doi: 10.1128/mBio.01114-17 (PMC5587909; doi:10.1128/mBio.01114-17)
Supplement: TABLE S1 [file mbo004173472st1.docx]

# Supplementary Table 1

Supplementary Table 1

| Gene | |  | MIC (µg/ml) |
| --- | --- | --- | --- |
| Name | Function |  | Polymyxin B |
| WT | Wild type |  | 8-16 |
| *atpA* | ATP synthase F1, alpha subunit |  | 2-4 |
| *vraG* | ABC transporter, permease protein |  | 1.5-2 |
| *vraF* | ABC transporter, ATP-binding protein |  | 1.5-2 |
| *graR* | DNA-binding response regulator |  | 1-2 |
