# Supplementary material for: Inhibition of the ATP Synthase Eliminates the Intrinsic Resistance of Staphylococcus aureus towards Polymyxins
Source: mBio. 2017 Sep 5;8(5):e01114-17. doi: 10.1128/mBio.01114-17 (PMC5587909; doi:10.1128/mBio.01114-17)
Supplement: TABLE S2 [file mbo004173472st2.docx]

# Supplementary Table 2

Supplementary Table 2

| Gene | |  | MIC (µg/ml) |
| --- | --- | --- | --- |
| Name | Function |  | Polymyxin B |
| SA113 | Wild type |  | 512 |
| Δ*dltA* | D-alanine-activating enzyme |  | 48 |
| Δ*mprF* | Phosphatidylglycerol lysyltransferase |  | 384 |
